# MicroRNA profiling of the murine hematopoietic system

**DOI:** 10.1186/gb-2005-6-8-r71

**Published:** 2005-08-01

**Authors:** Silvia Monticelli, K Mark Ansel, Changchun Xiao, Nicholas D Socci, Anna M Krichevsky, To-Ha Thai, Nikolaus Rajewsky, Debora S Marks, Chris Sander, Klaus Rajewsky, Anjana Rao, Kenneth S Kosik

**Affiliations:** 1 Department of Pathology, Harvard Medical School, and CBR Institute for Biomedical Research, Boston, MA 02115, USA; 2 Department of Biology and Genetics of Medical Sciences, Universitá degli Studi di Milano, 20133 Milan, Italy; 3 Computational Biology Center, Memorial Sloan-Kettering Cancer Center, New York, NY 10021, USA; 4 Department of Neurology, Brigham and Women's Hospital and Harvard Medical School, Boston, MA 02115, USA; 5 Center for Functional Comparative Genomics, Department of Biology, New York University, New York, NY 10003, USA; 6 Department of Systems Biology, Harvard Medical School, Boston, MA 02115, USA; 7 Neuroscience Research Institute, University of California Santa Barbara, Santa Barbara, CA 93106, USA

## Abstract

**Background:**

MicroRNAs (miRNAs) are a class of recently discovered noncoding RNA genes that post-transcriptionally regulate gene expression. It is becoming clear that miRNAs play an important role in the regulation of gene expression during development. However, in mammals, expression data are principally based on whole tissue analysis and are still very incomplete.

**Results:**

We used oligonucleotide arrays to analyze miRNA expression in the murine hematopoietic system. Complementary oligonucleotides capable of hybridizing to 181 miRNAs were immobilized on a membrane and probed with radiolabeled RNA derived from low molecular weight fractions of total RNA from several different hematopoietic and neuronal cells. This method allowed us to analyze cell type-specific patterns of miRNA expression and to identify miRNAs that might be important for cell lineage specification and/or cell effector functions.

**Conclusion:**

This is the first report of systematic miRNA gene profiling in cells of the hematopoietic system. As expected, miRNA expression patterns were very different between hematopoietic and non-hematopoietic cells, with further subtle differences observed within the hematopoietic group. Interestingly, the most pronounced similarities were observed among fully differentiated effector cells (Th1 and Th2 lymphocytes and mast cells) and precursors at comparable stages of differentiation (double negative thymocytes and pro-B cells), suggesting that in addition to regulating the process of commitment to particular cellular lineages, miRNAs might have an important general role in the mechanism of cell differentiation and maintenance of cell identity.

## Abstract 2

The first report of systematic miRNA profiling in cells of the hematopoietic system suggests that, in addition to regulating commitment to particular cellular lineages, miRNAs might have a general role in cell differentiation and cell identity.

## Background

MicroRNAs (miRNAs) represent a recently discovered class of small, noncoding RNAs, found in organisms ranging from nematodes to plants to humans. Many individual miRNAs are conserved across widely diverse phyla, indicating their physiological importance. The primary transcript (pri-miRNA) is generally transcribed by RNA polymerase II; it contains a typical stem-loop structure that is processed by a nuclear enzyme complex including Drosha and Pasha, and releases a 60- to 110-nucleotide pre-miRNA hairpin precursor [1]. The pre-miRNA is further processed by Dicer to yield the 19- to 22-nucleotide mature miRNA product, which is then incorporated into the RNA-induced silencing complex (RISC) [2-4]. RISC-bound miRNAs direct the cleavage and/or translational repression of messenger RNAs, thus providing post-transcriptional control of gene expression.

Like many transcription factors, miRNAs are important determinants of cellular fate specification. One of the most prominent and genetically best-studied examples is given by miRNAs involved in neuronal fate determination in *Caenorhabditis elegans*, where a cascade of several miRNAs and transcription factors regulate each other's activity to induce a different spectrum of putative chemoreceptors in the two main taste receptor neurons in *C. elegans *[5]. Furthermore, many miRNA genes are located at fragile sites, minimal loss of heterozygosity regions, minimal regions of amplification, or common breakpoints in human cancers, suggesting that miRNAs might play an important role in the pathogenesis of human cancer [6,7].

Hundreds of miRNAs have been identified in plants and animals, either through computational searches, RT-PCR-mediated cloning, or both. More than 200 human and rodent miRNAs have been reported and tabulated in the miRNA Registry [8], accounting for an estimated 1-2% of expressed human genes. Recent evidence suggests that the actual number of miRNAs is likely to be even larger [9,10]. MiRNAs have been implicated in biological processes ranging from cell proliferation and cell death during development to stress resistance, fat metabolism, insulin secretion and hematopoiesis [11]. However, for the most part, the regulation and function of most mammalian miRNAs are unknown. The bulk of the existing data on miRNA expression in mammalian cells has been derived from studies on whole tissues, which contain many heterogeneous cell types, or on transformed or established cell lines that may have diverged significantly from the primary cell types that they are assumed to represent [7,12-15]. To understand the role of miRNAs in mammalian development and differentiation, an important starting point is a systematic compilation of miRNAs expressed in individual cell types, especially those derived by differentiation from a common precursor.

The cells of the immune system originate from hematopoietic stem cells in the bone marrow, where many of them also mature. The hematopoietic stem cells give rise to both myeloid and lymphoid progenitors. The myeloid progenitor is the precursor of granulocytes, macrophages, dendritic cells, and mast cells of the innate immune system. Mast cells, whose blood-borne precursors are not well defined, terminate their differentiation in the body tissues, where they are widely distributed and where they orchestrate allergic responses and play a part in protecting mucosal surfaces against pathogens [16]. The common lymphoid progenitor gives rise to B and T lymphocytes and to natural killer cells. B lymphocytes differentiate in the bone marrow and T lymphocytes in the thymus; the stages of B and T cell development are defined by sequential rearrangement and expression of heavy- and light-chain immunoglobulin genes and TCR α and β chains, respectively. Mature B and T lymphocytes that have emigrated to the peripheral lymphoid organs, including the spleen and lymph nodes, but have not yet encountered their specific antigen are called 'naïve'. In the event of an infection, T lymphocytes that recognize the infectious agent are arrested in the lymphoid organs, where they proliferate and differentiate further into effector cells capable of combating the infection.

Because of the wealth of information available about the transcriptional and cellular networks involved in hematopoietic differentiation, the hematopoietic system is ideal for studying cell lineage specification. Many of the common progenitors of hematopoietic cells can be obtained as primary cells from humans and mice, and expanded and differentiated *in vitro*. Here we have performed a detailed analysis of miRNA expression in diverse hematopoietic cell types from the mouse, using a high-throughput system that allows analysis of many samples with minimal manipulation of the samples themselves. This has allowed us to identify miRNAs that are highly expressed in the hematopoietic system. Our results are consistent with a model of hematopoiesis in which transcriptional regulators act in concert with differentially expressed miRNAs to modulate the levels of mRNAs that control cell differentiation pathways.

## Results

### Microarray design

To probe the expression of miRNAs in a variety of different related and unrelated cell types, we chose to use miRNA arrays in preference to time-consuming Northern analysis that cannot be used efficiently with many different probes and samples. In the past year, several microarray methods have been developed [7,12,13,15,17-19]. Some of these groups [7,12,13,15,17] used cDNA or cRNA generated from total cellular RNA to apply to their microarrays. Other methods [18,19] rely heavily on several enzymatic steps, such as RNA ligation [18], or Klenow synthesis and exonuclease I degradation of ssDNA [19]. Instead, we chose a technique that does not involve reverse transcription of RNA and relies on only one enzymatic step ([20]; see Methods) thus reducing RNA manipulation to a minimum. In designing the arrays, we expanded the array dataset already developed by Krichevsky *et al*. [20]. The new generation of arrays contains 181 gene-specific oligonucleotide probes, corresponding to human, rat, and mouse miRNAs as reported in the miRNA Registry [8].

### Data from the arrays

**Figure 1:**
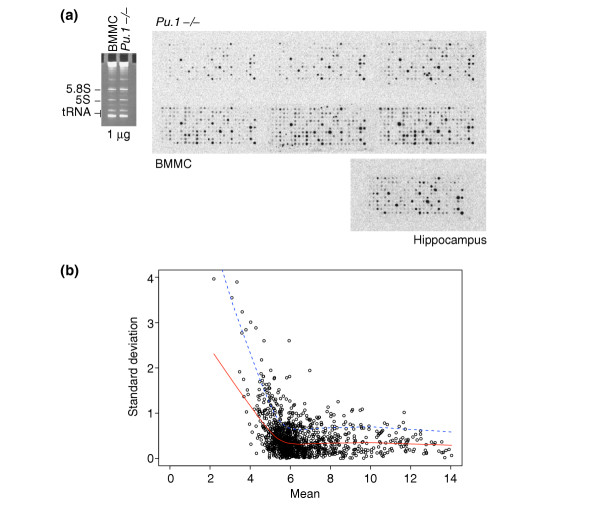
Design and reproducibility of microarrays. **(a) **Examples of microarrays: three membranes were used for each biological sample; arrays for *Pu.1-/- *cells, BMMC and hippocampus are shown. On the left, ethidium bromide staining of total RNA run on a denaturing gel for RNA quantification and quality control. **(b) **Plot of the standard deviation over replicates versus the mean of each replicate. The red line is a lowest fit to the distribution and the blue dotted line is twice the value of the red one. Points below the blue curve are considered good replicates; those above it are filtered out as too noisy. For this dataset, 86% of the spots were considered as good. BMMC, bone marrow-derived mast cells.

**Figure 2:**
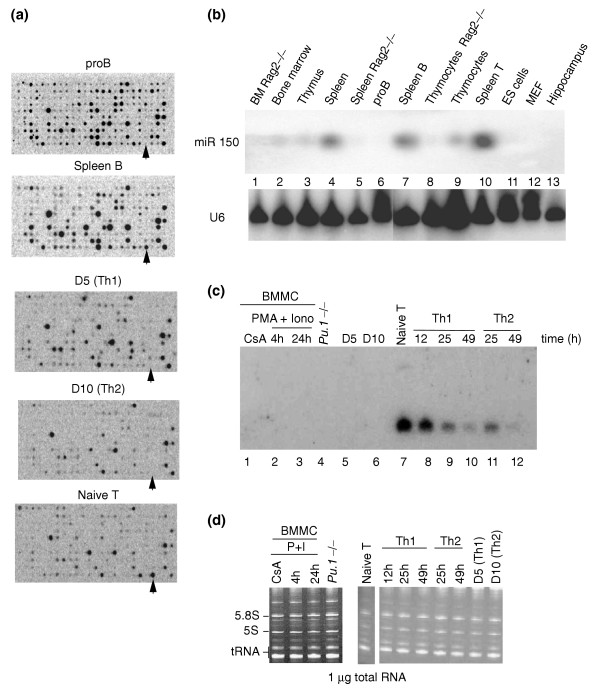
Comparison of miR-150 expression by arrays and Northern blotting. **(a) **Array data show that miR-150 (arrows) is highly expressed in spleen B and naïve T cells, but not in pro-B cells or fully differentiated Th1 and Th2 clones. **(b) **Northern blot analysis for miR-150 in various lymphoid and non-lymphoid tissues and cell types. U6 RNA levels are shown as loading control. **(c) **Northern blots of different cell types unstimulated or stimulated for the indicated amounts of time with either PMA and ionomycin (BMMC) or anti-CD3 and anti-CD28 (Th1 and Th2 primary cells). Preliminary data obtained both in Northern blot and arrays showed no difference in miRNA expression between BMMC left untreated or treated with cyclosporin A (CsA) (not shown). **(d) **Ethidium bromide staining of gel of total RNA from samples used in Figure 2c, showing equivalent RNA amounts. BMMC, bone marrow-derived mast cells; MEF, mouse embryo fibroblast.

Figure 1a shows a typical array experiment comparing miRNA expression in bone marrow-derived mast cells (BMMC) and a hematopoietic progenitor cell line (*Pu.1-/-*) derived from mice lacking the Ets-family transcription factor PU.1 [21]. This cell line differentiates efficiently into mast cells when rescued with PU.1 under conditions where GATA2 expression is maintained; expression of PU.1 in the absence of GATA2 results in commitment to the macrophage lineage instead [22]. Visual comparison of three arrays performed with each RNA sample shows the high reproducibility of the arrays, and emphasizes the difference in miRNA expression relative to hippocampal RNA (Figure 1a). This high degree of reproducibility was maintained over a total of nine arrays, each performed in triplicate using three independent RNA samples (not shown). Statistical analysis confirmed the high level of reproducibility (Figure 1b): when the standard deviation over replicates was plotted versus the mean of each replicate, 86% of the spots were considered good (see legend to Figure 1b for details). RNA loading for all arrays and Northern blot experiments was evaluated by ethidium bromide staining of a denaturing acrylamide gel as shown in Figures 1a and 2d.

The arrays were repeated using cells from several stages of lymphocyte differentiation (Figure 2a). Among the cell types compared were pro-B cells, which are in the process of rearranging the heavy-chain immunoglobulin locus; mature splenic B cells, which express IgM and IgD B cell receptors and are competent to respond to antigen; double-negative thymocytes (DN T), which are just beginning to rearrange their T cell receptor chains and lack surface expression of the CD4 and CD8 co-receptors; naïve CD4 T 'helper' cells, which have exited the thymus, bear the CD4 co-receptor and a mature T cell receptor, and are fully capable of recognizing and responding to antigen; and Th1 and Th2 T helper subsets, which are derived by differentiation from a common precursor, the naïve CD4 T cell, and are characterized by selective expression of the cytokines IFNγ and IL4, respectively. Figure 2a shows representative array data for pro-B cells, mature splenic B cells, naïve T cells, and Th1 and Th2 clones. Note that miR-150 is highly expressed in B cells purified from mouse spleen, but not in pro-B cells isolated from bone marrow of Rag2-/- mice; it is also expressed in naïve T cells but is down-regulated in the Th1 and Th2 T cell clones (Figure 2a, arrow).

### Validation of array data by Northern analysis

**Figure 3:**
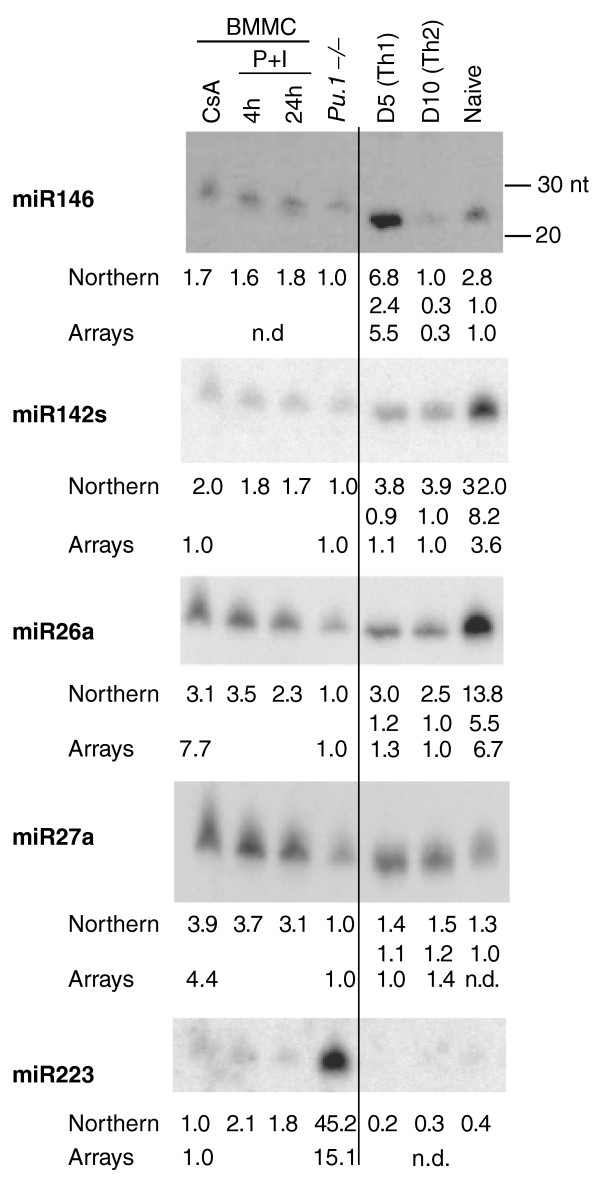
Microarrays and Northern blots correlate qualitatively and quantitatively. Northern blots for miRNA expression in mast cells (left panels), or T cells (right panels). BMMC were treated with cyclosporin A or PMA and ionomycin for the indicated amounts of time. Loading control is the same as Figure 2d. First row underneath the panels: ratio between the intensities of the Northern blot bands as assessed by phosphorimager and quantified by ImageQuant; all the samples are compared with *Pu.1-/- *cells except for miR-223, where each sample is compared with BMMC. Second row: these are also ratios between the intensities of the Northern blot bands, but the T cell samples are compared directly to each other. This allows a better direct comparison with the numbers on the third row, which are the ratios of BMMC versus *Pu.1-/- *cells (left panels) and D5 versus D10 versus naïve T cells as obtained from the arrays (right panels). BMMC, bone marrow-derived mast cells; CsA, cyclosporin A; n.d., not detectable.

**Figure 4:**
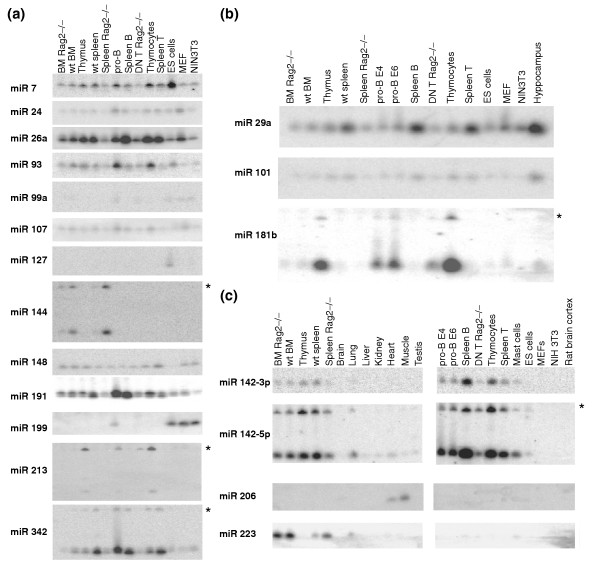
Additional Northern blots showing miRNA expression in various tissues and cell types. Shown are Northern blot data for the indicated miRNAs in different unstimulated cell types. Asterisks indicate bands of the size of pre-miRNA (60-70 nucleotides), which were detected in Northern blot for only a subset of the miRNAs analyzed. There was a good correlation overall between Northern blot data and expression data from the arrays (see heat map in Figure 5 and Table 2), with some exceptions, as discussed in the text. BMMC, bone marrow-derived mast cells; DN T, double-negative thymocyte.

Before analyzing the entire dataset from the microarrays, we validated the array results by Northern blot analysis. Single-stranded DNA oligonucleotides complementary to over 40 different miRNAs were used as probes; they were chosen because they were expressed in at least one cell type in the hematopoietic lineages and/or were highly expressed in neurons. Several of these Northern blots were already published with the first description of the microarray methodology [20] to confirm the array specificity. We performed several other Northern blots that included hematopoietic cells and tissues. These data are shown in Figures 2, 3 and 4, and summarized in Tables  1 and 2. With minor exceptions as discussed below, the results of Northern analysis were consistent with the array data for most miRNAs, by simple visual inspection (Table 2), and when the hybridization signal intensity was quantified by phosphorimager (Figure 3 and Table 1). For example, Northern analysis of miR-150 expression confirmed its expression in spleen B but not pro-B cells (Figure 2b, lanes 6 and 7), and in naïve T cells but not the Th1 and Th2 clones, D5 and D10 (Figure 2c, lanes 5-7). Figure 2b also shows that miR-150 is expressed in thymocytes and splenic T cells (lanes 9 and 10), but not in ES cells, mouse embryo fibroblasts or hippocampus (lanes 11-13). The lack of expression in *RAG2-/- *spleen and thymus (lanes 5 and 8) confirms that expression in these organs is confined to T and B lymphoid-lineage cells, and that within these lineages, miR-150 expression is restricted to cells that have developed beyond the DN T and pro B stages of development. Figure 2c confirms that naïve T cells show high level expression of miR-150 (lane 7) whereas the precursor cell line *Pu.1-/- *and BMMC, which are of the myeloid lineage, do not (lanes 1-4). Equivalent RNA loading in all lanes was confirmed by ethidium bromide staining of a denaturing acrylamide gel as shown in Figure 2d.

Strikingly, miR-150 expression in naïve T cells is rapidly down-regulated upon TCR engagement, regardless of whether T cells are stimulated under Th1 or Th2 conditions (Figure 2c, lanes 8-12). The levels of expression of miR-150 were already reduced by ~50% after 12 h of stimulation with plate-bound αCD3 and αCD28 (lane 8), and by >90% after 25 h (lanes 9 and 11), indicating a rapid and highly inducible mechanism of down-regulation. Expression was barely detectable after 49 h (lanes 10 and 12) and remained undetectable 3 days after stimulation (data not shown). Furthermore, miRNA expression was extinguished in fully committed Th1 and Th2 T cell clones (lanes 5 and 6). Together, these results suggest a role for miR-150 either in maintaining the undifferentiated status of naïve T cells or in promoting early steps in T cell differentiation.

Figure 3 extends the concordance of the array data with the Northern analysis to five additional miRNAs, emphasizing the cell type-specific changes that take place during differentiation. In the left panels of Figure 3, miRNA expression in BMMC treated with cyclosporin A to prevent activation, or stimulated with PMA and ionomycin for the indicated times, is compared with expression in the *Pu.1-/- *'precursor' cell line, which gives rise to mast cells when reconstituted with both PU.1 and GATA2 [22]. Three very different patterns are observed, exemplified by: miR-146 and 142s, which are expressed at essentially equivalent (low) levels in both the *Pu.1-/- *precursor cells and the fully-differentiated BMMC; miR-26a and 27a, which are expressed at low levels in the *Pu.1-/- *precursor cells and at three- to fourfold higher levels in fully differentiated BMMC; and miR-223, which is most highly expressed in the *Pu.1-/- *precursor cells and is barely detectable in the differentiated BMMC. The results of Northern analysis for these two cell types show an overall good concordance with the values obtained from the arrays (Figures 2, 3 and 4 and Table 1).

The right panels of Figure 3 compare expression of the same miRNAs in naïve T cells and fully-differentiated Th1 and Th2 cells (the D5 and D10 clones). Several expression patterns are evident: miR-146 is highest in Th1 cells and low in naïve T cells and Th2 cells; miRNAs 142s and 26a are expressed at higher levels in the precursor naïve T cells; miR-27a is equivalently expressed in both the precursor naïve T cells and the differentiated Th1 and Th2 cells; and miR-223 is very poorly expressed in all these T cell types. The relative expression of these miRNAs in naïve versus differentiated T cells was confirmed in primary cultures of Th1 and Th2 cells (see Table 1). There was full concordance of the Northern analysis with the miRNA array data for miRNAs 146, 142s, 26a and 223 (Figure 3, Table 1); however, as discussed further below, the signal for 27a and a handful of other miRNAs expressed at low levels in naïve T cells fell below the limit of detection on the microarrays.

**Figure 5:**
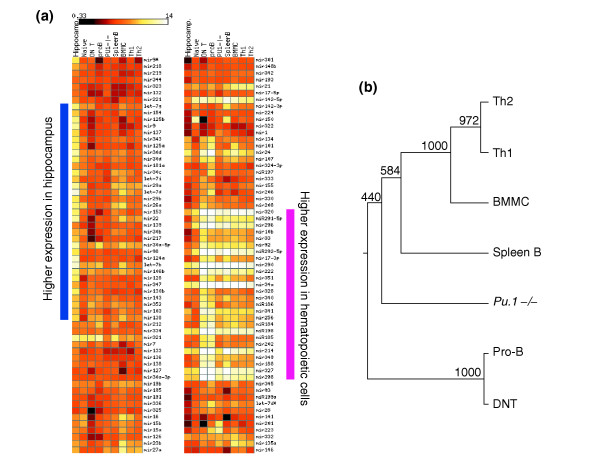
Analysis of microarray data. **(a) **Heat map of miRNAs expressed at least three times over the background for at least one of the samples. **(b) **Hierarchical clustering of hematopoietic samples (see analysis in Table 3). DN T, double-negative thymocyte; MEF, mouse embryo fibroblast.

Table 1 summarizes the results from Northern analysis of the miRNAs shown in Figures 2 and 3, as well as showing data for two additional miRNAs, let7d (let7 family) and miR-222. The miRNA expression pattern of D5 and D10 T cell clones was comparable with that of differentiated primary Th1 and Th2 cells respectively, validating the use of D5 and D10 cells as models for fully differentiated Th1 and Th2 cells. Like miR-150, the expression of miR-142s, miR-26a and let7d showed a rapid decline during differentiation of naïve T cells into Th1 or Th2 effectors. miR-27a was expressed at equivalent levels in naïve and differentiated T cells. miR-146 showed a Th1-skewed expression pattern: it's levels increased in Th1 cells and decreased in Th2 cells relative to it's expression in naïve T cells. We have not yet detected an miRNA with the converse expression pattern of high expression in Th2 cells relative to Th1. miR-222 was detectably expressed in BMMC, *Pu.1-/- *precursor cells and fully differentiated Th1 cells, but it's expression was not detectable in the other cell types tested (Table 1); in contrast, miR-16 was expressed in all cell types analyzed, but it's expression was relatively variable both in arrays and Northern blots, so quantification was not attempted (data not shown). Some of our data confirm published reports. For example, miR-223 is reported to be expressed in myeloid cells [7,23]; miR-125 and 128 are highly expressed in the brain [13,14]; and miR-16 is expressed in a wide variety of tissues [7,14,23] (see also heat map of expression in Figure 5a).

Figure 4 shows Northern blot data for additional miRNAs. Most of the data from Northern blots correlated at least qualitatively with the expression data from the arrays (Table 2; also compare data in Figures 3 and 4 to the heat map in Figure 5). Some exceptions were noted. For some of the miRNAs (miR-129, 151, 184, 185, 202, 212 and 351), we could not obtain any hybridization signal on Northern blots, so we were unable to compare Northern and array data. MiR-223 and miR-206 showed poor correlation between the arrays and the Northern blots: for miR-223, we detected a higher level of expression in pro-B in the arrays compared with what we detected on Northern blot, while for miR-206, the arrays showed high expression in pro-B and DN T that was undetectable by Northern blot. In a few other cases, the hybridization signal was lower in the arrays compared with Northern blots, but the relative expression levels between different cell types was similar. It is unclear at this point why the expression of some miRNAs appears different depending on the method used to detect them.

In a few cases, the probes used in Northern analysis hybridized to a cross-reacting band with a molecular weight higher than the mature or pre-miRNA molecules. In these cases the correlation between Northern blots (which use total RNA) and arrays (which use the low-molecular weight RNA) was only partial. Even though our system is designed to exclude RNA molecules bigger than ~300 nucleotides, we effectively obtained exclusion of molecules bigger than 60-80 nucleotides (as shown in [20]). Thus, the changes observed mainly reflect changes in mature miRNA levels, as also shown by the correlation with Northern blots. However, it remains possible that a strong expression of cross-reacting RNA close to this size might partially alter the array results; we observed such bands for miR-186, miR-188 and miR-321. Of note, miR-321 has been removed from the microRNA Registry because it was identified as a fragment of an Arg tRNA and not a miRNA.

Despite differences in methods as well as in the number of microRNAs analyzed, there is good agreement between our results and those of others, with regard to specificity and sensitivity, when array and Northern blot analyses are examined for similar cell types [13,14]. Similar to the findings and discussion of Miska *et al*. [13], we do not expect our microarray technique to provide sufficient specificity to distinguish reliably between hybridizing sequences that have only one or few nucleotide mismatches. Although hybridization signals from several control probes containing three staggered nucleotide mismatches were lower than that for the corresponding miRNA probes (see also Material and methods), our method cannot efficiently discriminate between close miRNA paralogs. This limitation is alleviated somewhat by the fact that for most miRNAs, the most closely related paralogs differ by five mismatches or more [13]. The sensitivity of the arrays is similar to that of Northern blots. Synthetic RNA oligonucleotides 'spiked' into cellular RNA samples prior to array hybridization were detected at a 2-20 fmole range. Northern blot allowed detection of as little as 1-10 fmole of synthetic oligonucleotides (data not shown). In summary, therefore, we saw substantial concordance between arrays and Northern blots, allowing us to identify cell type-specific differences in miRNA expression as well as differences between miRNAs expressed by precursor cells and their differentiated progeny. This led us to analyze the array data more extensively using computational methods.

### Analysis of miRNA arrays

To identify patterns of miRNA expression among the cell types tested, we arranged the array data for miRNAs that were expressed at least three times over the background for at least one of the samples in a heat map (Figure 5a). Brown to white colors indicate increasing levels of miRNA expression in arrays. This analysis revealed a cluster of miRNAs that were preferentially expressed in the hippocampus compared with hematopoietic cells, as indicated by the blue bar in the left panel. MiRNAs expressed at higher levels in the hematopoietic system are indicated by the purple bar in the right panel.

To achieve a better understanding as to how miRNA expression patterns correlate with hematopoietic cell differentiation, we performed a hierarchical clustering of the normalized array data for hematopoietic cell types (Figure 5b). The subset of miRNAs detected in at least one hematopoietic cell sample was used to compute the distance function from the Pearson correlation between samples (Table 3). Standard hierarchical clustering with average linkage was used, and bootstrap resampling was employed to assess the robustness of the clustering results. This analysis showed that fully differentiated effector cells (Th1, Th2 and BMMC) are more closely related to each other in their miRNA expression pattern than to their respective precursor cells (DN T and *Pu.1-/- *precursor cells). The miRNA expression patterns of pro-B and DN T, precursor cells for the B and T lymphocyte lineages respectively, were also very closely related. Although the detected miRNA expression pattern of naïve T cells most closely resembled that of splenic B cells (Table 3), naïve T cells were excluded from the clustering analysis. This was because RNA isolated from naïve T cells yielded much lower overall array hybridization signals compared with RNA from the other cell types examined, causing the signal for a handful of expressed miRNAs to fall below the limit of detection for the microarrays (for example, miR-27a, see Figure 4 and Table 1), and making it impossible to accurately normalize the array data for naïve T cells relative to the signal obtained from other cell types.

## Discussion

**Figure 6:**
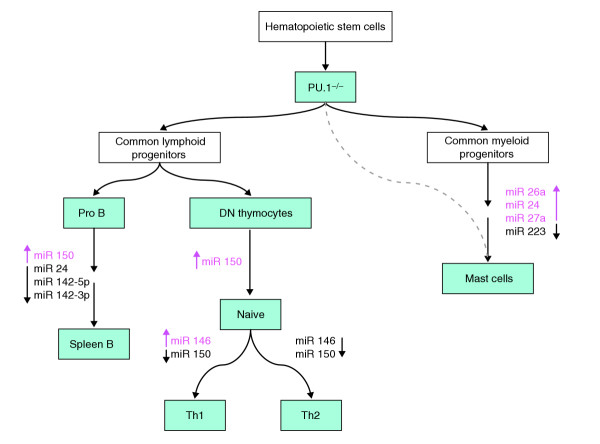
MiRNA expression and lineage commitment. Expression of miRNA in the hematopoietic system changes depending on the differentiation status. Part of the hematopoietic differentiation tree is represented: myeloid and lymphoid progenitors derive from a common progenitor, which is represented by the model *Pu.1-/- *cell line (see text). The common lymphoid progenitor gives rise to B and T lymphocytes and the common myeloid progenitor gives rise to mast cells and other cells types. Superimposed on this diagram are examples of miRNAs that were discovered to be differentially expressed between the indicated precursor/progeny pairs using array analysis with confirmation by Northern blot. DN, double-negative.

In summary, pairwise comparisons of the expression of 181 mature miRNAs in selected highly purified hematopoietic cell types at immature, mature, and effector stages revealed specific differences between related cell types (see also Additional Data Files 1, 2, 3). As described above, the differences were confirmed by Northern analyses (Figures 2, 3 and 4, and Tables 1 and 2) and revealed a subset of miRNAs expressed at higher levels in the hematopoietic system compared with neuronal tissue (Figure 5a). Figure 6 shows a schematic hematopoietic lineage tree, where lymphoid and myeloid cells derive from a common lymphoid and common myeloid progenitor, respectively. Both these cell types derive from a common precursor further upstream in the differentiation process and we used the *Pu.1*-/- cells as a model for such precursors. The figure also shows a summary of some confirmed changes in miRNA expression in the different stages of cell differentiation superimposed on the diagram, showing precursor-progeny relationships in lymphocyte and mast cell differentiation. Even though only selected hematopoietic cell lineages were analyzed, each differentiation step was characterized by changes in miRNA expression, with some miRNAs showing increased and some showing decreased expression. MiR-150 expression is of particular interest: this miRNA is up-regulated during the developmental stages of B and T cell maturation, but down-regulated again during the further differentiation of naïve T cells into effector Th1 and Th2 cells. miR-146 is also notable, since it is upregulated in Th1, but not Th2 cells. We therefore predict that these miRNAs probably play a role in establishing and/or maintaining cell identity in lymphocytes.

Differentiation of naïve T cells into Th1 and Th2 effector cells is a particularly tractable system for studying cell lineage specification. Northern blot analyses indicated that several miRNAs are rapidly down-regulated following activation of naïve T cells under both Th1 and Th2 differentiation conditions. miR-146 was a clear exception to this pattern, being up-regulated in Th1, but not Th2 cells. In this respect, *miR-146 *joins a group of Th1-associated genes that include cytokines (*Ifnγ*, *Tnfα*), chemokine receptors (*Cxcr3*, *Ccr5*), and transcription factors (*Tbx21*, *Hlx*) [6,24,25]. Our results suggest two hypotheses for further testing. Firstly, the transcription of pri-miRNAs may be controlled by the same transcription factors known to control specific cell differentiation events. For example, T-bet directs *Ifnγ *expression, and may also activate transcription of *pri-miR-146*. Conversely, miRNAs that are regulated during cell differentiation may target one or more of the mRNAs known to be differentially expressed between Th1 and Th2 cells. Although the predicted targets of conserved miRNAs represent a diverse array of gene products [26-30], targeting of key transcription factors would represent a particularly efficient means for miRNA participation in cell fate decisions.

Our results call to attention the 'common logic' and shared roles of transcriptional regulation by transcription factors and post-transcriptional regulation by miRNAs [5]. It is likely that both pathways of regulation are integral to successful regulation of hematopoietic cell differentiation. Decades of research have been devoted to the elucidation of the transcriptional networks involved in hematopoiesis. For example, the Ets family transcription factor, PU.1, is necessary for the generation of certain hematopoietic lineages but not others: it directs the differentiation of hematopoietic progenitors into macrophages, neutrophils, B lymphocytes and mast cells, but is not involved in erythroid or megakaryocytic differentiation [21,22]. Lineage specification is controlled by the level of PU.1 expression and by which partner transcription factors are co-expressed: for instance, low levels of PU.1 and co-expression of early B-cell factor (EBF) control differentiation to the B cell lineage, whereas high levels of PU.1 predispose to macrophage differentiation unless GATA2 is co-expressed, in which case differentiation is tilted to the mast cell lineage [22,31,32]. It will be informative to compare miRNA expression in *Pu.1-/- *progenitor cells that have been reconstituted to promote differentiation along these various lineages. Further study of the expression and function of miRNAs in hematopoiesis will probably uncover additional complexity and subtlety, as well as interconnections between miRNA and transcription factor networks [5].

Systematic analysis of miRNA expression patterns within our dataset indicates that besides influencing the process of commitment to a particular cellular lineage, miRNAs may play an important general role in the mechanism of cell differentiation and maintenance of cell identity. The highest degree of correlation in the expression pattern of miRNAs was observed 'horizontally' between hematopoietic cell types at a similar stage of differentiation. For instance, early B and T cell precursors are more closely related to each other in their miRNA expression patterns than to their more differentiated progeny, mature splenic B cells and naïve T cells. Most strikingly, fully differentiated effector cells, including the closely related Th1 and Th2 cells, but also the much more distantly related BMMC, are more closely related to each other in their miRNA expression pattern than to their respective precursor cells. The high degree of correlation in the miRNA expression patterns of these distantly related immune effectors suggests that a common set of miRNAs may be employed in both lineages to regulate similar effector functions, such as tissue homing and cytokine production. Alternatively, these findings may reflect a general role for some of these miRNAs in stabilizing gene expression and thereby lineage specification. This could be accomplished through the promiscuous targeting of many transcripts or by specific targeting of genes that regulate the plasticity of transcriptional states, such as chromatin-modifying proteins. Similarly, miRNAs shared among early precursors may regulate precursor cell self-renewal and maintenance of an undifferentiated state. The rapid loss of several miRNAs early in the process of differentiation of Th1 and Th2 effector cells from naïve T cell precursors is consistent with this concept.

## Conclusion

We report miRNA expression patterns for diverse murine hematopoietic cells types, identify a subset of miRNAs preferentially expressed in the hematopoietic system compared with neuronal cells, and identify individual miRNA expression changes that occur during cell differentiation. Our data support the use of the miRNA microarray for detection of patterns of miRNA expression and for quantification of miRNA expression, with the obvious advantage that the expression of several hundred genes can be identified in the same sample at once, and with relatively small amounts of total RNA. Deciphering the miRNA expression status of cells under different conditions of development and activation and in different disease states will be useful to identify miRNA targets, and alterations in the pattern of miRNA expression may disclose new pathogenic pathways and new ways to target diseases.

## Materials and methods

### Tissue preparation, cells differentiation and RNA extraction

Hippocampi were dissected from 10-14 week old Balb/c mice. For pro-B cell preparation, bone marrow cells were isolated from femurs and tibias of 6-12 week old *Rag2-/- *C57BL/6 × 129 mice and pro-B cells were isolated using CD19 MACS beads. Spleen B cells were isolated using CD19 MACS beads from splenocytes obtained from 6-12 week old C57BL/6 mice. Double-negative thymocytes (DN T) were obtained from the thymi of 4-6 week old *Rag2-/- *C57BL/6 × 129 mice without purification; more than 90% of the cells were DN2 and DN3 (not shown). Due to the low amount of cells that can be obtained from one mouse, proB and DN T cells were purified from 20 *Rag-/- *mice, while the spleen B cells were obtained from 10 C57BL/6 mice, and the samples were pooled.

BMMC preparation and differentiation was as previously described [33,34]. Briefly, bone marrow cells were isolated from femurs and tibias of 6-12 week old BALB/c mice and maintained for 4-12 weeks in RPMI medium containing 50% WEHI-3 (American Type Culture Collection, VA, USA) conditioned supernatant as a source of IL-3.

*Pu.1 *knock-out cells were kindly provided by Dr Harinder Singh (University of Chicago), and were maintained in IMDM media supplemented with 10 ng/ml of recombinant IL-3 (Peprotech Inc., NJ, USA). Naïve CD4 cells were purified from spleen and lymph nodes of *Tcrα-/- *DO11 TCR transgenic mice by magnetic bead selection (Dynal, Oslo, Norway) as previously described [33]. Primary Th1 and Th2 cells were differentiated in culture for 7 days as previously described [35]. Murine Th1 (D5) and Th2 (D10) clones were maintained as previously described [36]. RNA was prepared using Ultraspec or Trizol reagents following manufacturer's instructions.

### Oligonucleotide array for miRNA

This method has been previously described (see [20]). Briefly, trimer oligonucleotides (antisense to miRNAs) of 54-72 nucleotides at a final concentration of 7 μM were spotted on GeneScreen Plus (NEN) membranes with a 1536 pin plate replicator (V&P Scientific, CA, USA). Oligonucleotides were immobilized in 100 mM NaOH, after which the membranes were briefly neutralized in 5% SDS at room temperature and with 0.2% SDS at 72°C. Arrays were stored in 0.2% SDS at -4°C.

Total RNA (5-10 μg) from hippocampus tissue and various hematopoietic cell types was preheated at 80°C for 3 min, cooled on ice and filtered through Microcon YM-100 concentrators to obtain a low molecular weight (LMW) fraction of RNA enriched in molecules less than 60 nucleotides in size. The LMW RNA was end-labeled with 30 μCi of γ^33^P dATP (3000 Ci/mmole) with T4 polynucleotide kinase, and purified using the QIAgen Nucleotide Removal kit (QIAgen Inc., CA, USA).

For hybridization, membranes were first prehybridized in MicroHyb hybridization buffer (ResGen, AL, USA) at 37°C for at least 30 min, followed by an overnight hybridization in the same solution containing the RNA probe. Following hybridization, membranes were washed twice in 2 × SSC/0.5% SDS at 37°C and once in 1 × SSC/0.5% SDS at 37°C. Membranes were exposed to a phosphor storage screen, scanned using a Phosphor Imager, and signals were quantified using the ImageQuant software (Molecular Dynamics, CA, USA). For reuse, membranes were stripped with 0.2% SDS at 72°C, tested again by exposure to phosphorimager screen, and rehybridized three to five times. Each experiment included two to three independent RNA samples and to ensure accuracy of the hybridizations, each RNA sample was hybridized with three membranes.

To confirm specificity, a series of oligonucleotides with three mismatches (G>C or C>A) were included on the array. These mismatches resulted in a significant drop in signal intensity as compared with their cognates. The melting temperature of oligo probe:miRNA pairs could affect the sensitivity and specificity of the arrays for different miRNAs. An analysis of this correlation showed that hybridization signals significantly above background were obtained for probes in a wide range of melting temperatures (Additional Data File 4). Also, three synthetic 21-nt RNA oligonucleotides with sequences that do not correspond to any known miRNA, but that are exact complements to randomly spotted sequences, were added to the RNA samples at a known concentration as a reference for normalization.

### Northern blot analysis

Total RNA (20 μg) was loaded and separated on a denaturing 12-15% polyacrylamide gel and transferred electrophoretically to a GeneScreen Plus or Nytran SuPerCharge membrane (Scheicher and Schuell, NH, USA). Membranes were UV-crosslinked. Probes were prepared by T4 polynucleotide kinase labeling of antisense oligonucleotides with γ^32^P dATP. Hybridization was performed with UltraHyb Hybridization buffer (Ambion, TX, USA) or Denhardt's solution at 37-42°C. Blots were washed at the same temperature with 2 × SSC/0.1% SDS with a brief final wash with 0.1 × SSC/0.1% SDS. Radiolabeled Decade RNA markers (Ambion) were loaded as size markers. tRNA and 5S RNA stained with ethidium bromide served as a sample loading control. For reuse, blots were stripped by boiling in 0.1 × SSC/0.1% SDS twice for 10 min and reprobed.

### Data analysis

Before analysis, the raw data needed to be processed in order to handle overall scaling differences between the individual scans and negative values arising from the background subtraction. Although these are common issues in array-based experiments, it is not obvious what the optimal preprocessing algorithm should be. For the data presented here, the Variance Stabilization Normalization method [37] was used. The method has a considerable advantage in that it uses a generalized log transformation that can deal directly with negative values, eliminating the need to artificially shift or truncate these data points.

Standard hierarchical clustering with average linkage was used to cluster the hematopoietic samples. A subset of the miRNAs was used to compute the distance function. These miRNAs had to have a signal level of three times the background standard deviation in at least one of the hematopoietic samples. The Pearson correlation was used to compute the distance function with dist = (1-ρ)/2 where ρ is the correlation. To assess the robustness of the results, bootstrap resampling was carried out using a parametric method to add noise to the data. Gaussian noise with zero mean and a standard deviation equal to that for each spot's replicates was added to each point. One thousand resampled datasets were created and clustered with a consensus tree built from the results. The number at each node indicates how often that subtree appeared in the 1000 replica trees.

To identify miRNAs that were differentially expressed between the various sample subtypes, a variation of the standard *t*-test was used on the transformed expression values. To handle the low number of samples, a Bayesian correction method [38] was used to adjust the standard deviation. To account for the multiple testing problem, the False Discovery Rate (FDR) method was used and the lists were cut off at specific values of the FDR. Additionally, the results were filtered to include only those miRNAs that were expressed threefold above the background standard deviation in at least one sample.

### Data availability

The primary microarray data is deposited in the ArrayExpress database with accession number E-MEXP-372.

## Additional data files

The following additional data are available with the online version of this article: miRNAs differentially expressed between hippocampus and combined hematopoietic samples (Additional data file 1), miRNAs differentially expressed between BMMC and Th1, Th2 and *Pu.1-/- *cells (Additional data file 2), miRNAs differentially expressed between spleen B versus pro-B (Additional data file 3), and an analysis of the probe melting temperatures (Tm) versus the average signal obtained in the arrays (Additional data file 4).

## Supplementary Material

Additional data file 1 For normalization the VSN method was used [37]. A standard *t*-test on the generalized log transformed expression levels was used to rank the miRNAs and compute *p *values. The results were filtered to discard any miRNA whose signal was not three times above the background in at least one of the two groups and whose absolute fold change was less than 3. Click here for file

Additional data file 2 To handle the low sample numbers, the Bayesian corrected *t*-test [38] was used to rank and compute *p *values. The different comparisons had greatly varying significances, so different cut offs were used on each list. For the BMMC versus Th1 and Th2 comparisons, a FDR cutoff of 10^-2 ^was used, with a filter of three times background levels and a fold change of three. For the BMMC versus *Pu.1-/- *comparison a FDR of 10^-6 ^and four times background filter was used. Click here for file

Additional data file 3 The FDR cut off was 10^-6 ^and the background filter was four times. Click here for file

Additional data file 4 There is a clear correlation between Tm and strength of signal for all the probes used (left panel). The same is true if only the probes for miRNAs that were identified as 'high in hematopoietic cells' (see Figure 5a) are considered (right panel). Also, the latter probes have Tm that span almost the entire range of Tm seen for all the probes, and have an average Tm (56°C) only slightly higher than the average of all the probes (54°C). This means that overall, the use of these different oligos as probes in the arrays is in fact valid, even though we may have lost some of the low Tm miRNAs as false negative signals, and some of the high Tm probes may have given a few false positive signals. Click here for file

## Figures and Tables

**Table 1:** Northern blot quantification of miRNA

miR	BMMC	*Pu.1-/-*	Naïve	Th1 (49 h)	D5 Th1	Th2 (49 h)	D10 Th2
150	n.d.	n.d.	39.4	3.5	n.d.	1.0	n.d.
146	1.7	1.0	2.8	3.7	6.8	1.3	1.0
142s	2.0	1.0	32.0	7.5	3.8	4.7	3.9
26a	3.1	1.0	13.8	1.4	3.0	1.2	2.5
27a	3.9	1.0	1.3	0.9	1.4	0.8	1.5
223	1.0	45.0	n.d.	n.d.	n.d.	n.d.	n.d.
Let7d	2.3	1.0	4.7	2.1	2.5	1.7	1.7
222	1.0	1.2	n.d.	n.d.	1.1	n.d.	n.d.

Northern blots for miRNA expressed in mast cells, precursor cells, and T cells at various stages of differentiation were quantified by phosphorimager. BMMC, bone marrow-derived mast cell; DN T, double-negative thymocyte; n.d., not detectable.

**Table 2:** Correlation between arrays and Northern blot data

		DNT	Pro-B	Spleen B	BMMC	Hippocampus
MiR 7	Arrays	+	++	++	+/-	++
	Northerns	+	+++	++	n.a.	n.a.
MiR 24	Arrays	+++	++++	+++	++	++
	Northerns	++	++++	+++	n.a.	n.a.
MiR 26a	Arrays	+	+	++	+++	+++
	Northerns	+	++	+++	n.a.	n.a.
MiR 29a	Arrays	+	+	++	++	+++
	Northerns	+	+	++	nd	+++
MiR 93	Arrays	++	++	+/-	++	+/-
	Northerns	+	++	+	n.a.	n.a.
MiR 99a	Arrays	n.s.s.	n.s.s.	n.s.s.	n.s.s.	n.s.s.
	Northerns	+/-	+/-	-	nd	nd
MiR 101	Arrays	++	+++	+	+++	++
	Northerns	+	+	+	nd	++
MiR-107	Arrays	+++	+++	++	++	++
	Northerns	+	++	+	n.a.	n.a.
MiR-127	Arrays	+/-	+	+	+/-	+
	Northerns	-	-	-	n.a.	n.a.
MiR-142-3p	Arrays	+++	+++	++++	+++	+
	Northerns	++	+++	++++	++	n.a.
MiR-142-5p	Arrays	++	+	+++	++	+
	Northerns	+	++	+++	+	n.a.
MiR-144	Arrays	n.s.s.	n.s.s.	n.s.s.	n.s.s.	n.s.s.
	Northerns	-	-	-	n.a.	n.a.
MiR-148	Arrays	+	+	+	+	+/-
	Northerns	+	+	+	n.a.	n.a.
MiR-150	Arrays	-	+	+++	+	+/-
	Northerns	-	-	+++	n.a.	-
MiR-181b	Arrays	+	+	+	+	++
	Northerns	+	+	-	n.a.	+
MiR-191	Arrays	+/-	+	+	+	+
	Northerns	+	++	++	n.a.	n.a.
MiR-199	Arrays	+	+	+	+	+/-
	Northerns	-	+	-	n.a.	n.a.
MiR-206	Arrays	++	+++	+	+/-	+/-
	Northerns	-	-	-	-	n.a.
MiR-213	Arrays	n.s.s.	n.s.s.	n.s.s.	n.s.s.	n.s.s.
	Northerns	-	+/-	-	n.a.	n.a.
MiR-223	Arrays	+	+++	+	+/-	+
	Northerns	+/-	-	+	+	n.a.
MiR-342	Arrays	+	+	+	+	+
	Northerns	+	+++	++	n.a.	n.a.

The table summarizes and compares the Northern blot data shown in Figure 4 and the array data shown in the heat map in Figure 5. Northern blot and array data were scored independently using an arbitrary scale from undetectable (-) to strongly detected (++++) to indicate relative signal intensity in each case. BMMC, bone marrow-derived mast cell; DN T, double-negative thymocyte; n.s.s., non statistically significant hybridization signal; n.a., not analyzed.

**Table 3:** Correlation coefficients (Pearson correlation)

	Naïve	DN T	Pro-B	*Pu.1-/-*	Spleen B	BMMC	Th1	Th2
Naïve	1.00							
DN T	0.50	1.00						
Pro-B	0.47	0.94	1.00					
*Pu.1-/-*	0.56	0.83	0.78	1.00				
Spleen B	0.76	0.87	0.80	0.81	1.00			
BMMC	0.63	0.74	0.68	0.78	0.79	1.00		
Th1	0.68	0.85	0.79	0.84	0.88	0.92	1.00	
Th2	0.67	0.83	0.80	0.84	0.87	0.90	0.96	1.00

The table represents a standard statistical correlation between the indicated samples, where 1 = perfectly correlated and 0 = uncorrelated. BMMC, bone marrow-derived mast cell; DN T, double-negative thymocyte.
