# Supplementary figures and images for: MicroRNA profiling of the murine hematopoietic system
Source: Genome Biol. 2005 Aug 1;6(8):R71. doi: 10.1186/gb-2005-6-8-r71 (PMC1273638; doi:10.1186/gb-2005-6-8-r71)

**Tm vs. Ave signal (all probes)**

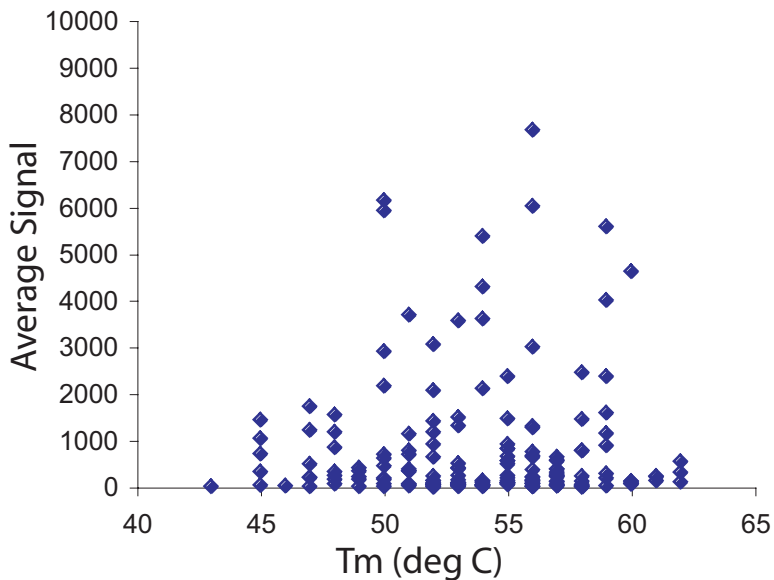

**Tm vs. Ave signal (high in hemat.)**

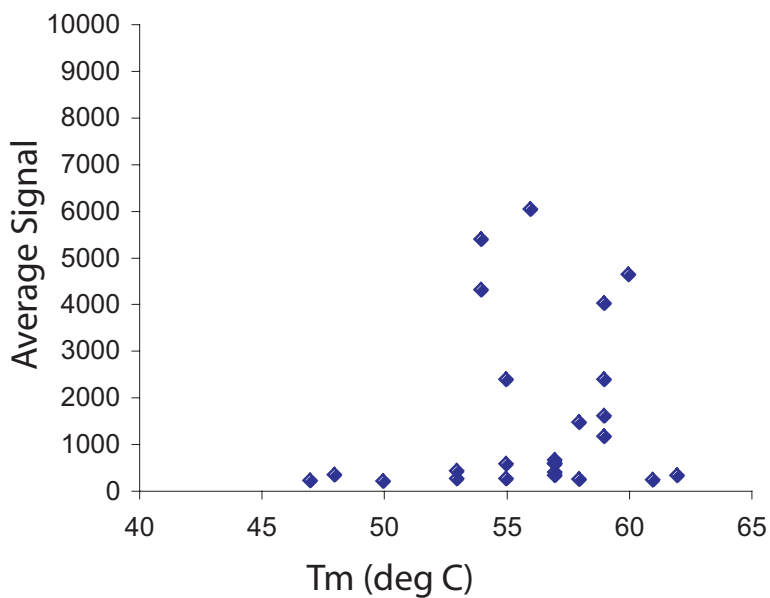

Supplement: Additional data file 4 — There is a clear correlation between Tm and strength of signal for all the probes used (left panel). The same is true if only the probes for miRNAs that were identified as 'high in hematopoietic cells' (see Figure 5a) are considered (right panel). Also, the latter probes have Tm that span almost the entire range of Tm seen for all the probes, and have an average Tm (56°C) only slightly higher than the average of all the probes (54°C). This means that overall, the use of these different oligos as probes in the arrays is in fact valid, even though we may have lost some of the low Tm miRNAs as false negative signals, and some of the high Tm probes may have given a few false positive signals. [file gb-2005-6-8-r71-S4.pdf]
