# Supplementary material for: MicroRNA profiling of the murine hematopoietic system
Source: Genome Biol. 2005 Aug 1;6(8):R71. doi: 10.1186/gb-2005-6-8-r71 (PMC1273638; doi:10.1186/gb-2005-6-8-r71)
Supplement: Supplementary file 4 [file gb-2005-6-8-r71-S1.doc]

Name	p-value	Mean.Brain	Mean.Hema	Fold Change
**mir9**	1.53E-48	960.38	9.82	97.83
**mir128**	2.95E-40	406.82	14.35	28.36
**mir9***	2.79E-35	354.53	12.84	27.61
**mir130b**	3.44E-35	377.07	12.97	29.07
**mir343**	5.71E-35	334.53	24.70	13.54
**mir125b**	2.93E-32	521.88	14.55	35.86
**mir124a**	7.48E-32	1174.05	12.77	91.97
**mir153**	1.30E-31	332.35	18.88	17.60
**mir30d**	7.59E-31	694.22	34.93	19.87
**mir137**	1.12E-30	206.60	11.54	17.90
**mir125a**	1.42E-29	303.80	14.70	20.67
**mir29a**	2.73E-28	908.82	57.44	15.82
**let-7d**	1.39E-27	1358.59	58.21	23.34
**mir98**	1.05E-26	258.23	15.76	16.38
**mir218**	1.26E-26	145.69	11.72	12.43
**mir142-5p**	3.94E-25	92.61	511.02	-5.52
**mir154**	6.06E-25	183.26	18.71	9.79
**mir29b**	1.28E-24	298.88	24.76	12.07
**let-7b**	2.79E-24	1015.41	52.57	19.32
**miR291-5p**	8.77E-24	21.65	211.02	-9.75
**let-7g**	1.64E-23	392.49	30.50	12.87
**miR292-5p**	3.05E-23	112.76	925.05	-8.20
**mir22**	1.70E-22	626.88	51.32	12.21
**mir30a-5p**	4.11E-22	730.32	82.77	8.82
**mir296**	1.33E-20	89.72	544.45	-6.07
**mir30c**	7.27E-19	274.01	33.44	8.19
**mir26a**	1.52E-18	466.33	57.95	8.05
**mir30b**	1.93E-18	146.25	21.11	6.93
**mir327**	1.97E-18	232.35	921.88	-3.97
**mir290**	5.36E-18	318.02	1652.92	-5.20
**mir143**	6.03E-18	151.14	23.34	6.47
**mir30d**	2.74E-17	252.51	23.38	10.80
**mir138**	8.90E-17	228.22	40.65	5.61
**mir106b**	1.08E-16	381.57	61.44	6.21
**mir352**	1.29E-16	182.63	38.60	4.73
**mir320**	1.56E-16	155.72	1136.97	-7.30
**let-7i**	1.41E-15	179.69	24.29	7.40
**mir92**	1.02E-13	55.45	175.33	-3.16
**miR198**	3.61E-13	247.19	761.34	-3.08
**mir34a**	6.69E-13	308.38	1868.27	-6.06
**mir349**	7.90E-13	31.92	96.94	-3.04
**mir222**	1.95E-12	163.93	495.81	-3.02
**mir101**	6.49E-11	145.64	48.06	3.03
**mir321**	6.20E-09	348.07	83.83	4.15
**mir214**	6.99E-08	116.79	571.27	-4.89
